# Supplementary material for: Polymorphisms in Human APOBEC3H Differentially Regulate Ubiquitination and Antiviral Activity
Source: Viruses. 2020 Mar 30;12(4):378. doi: 10.3390/v12040378 (PMC7232234; doi:10.3390/v12040378)
Supplement: Supplementary file 1 [file viruses-12-00378-s001.pdf]

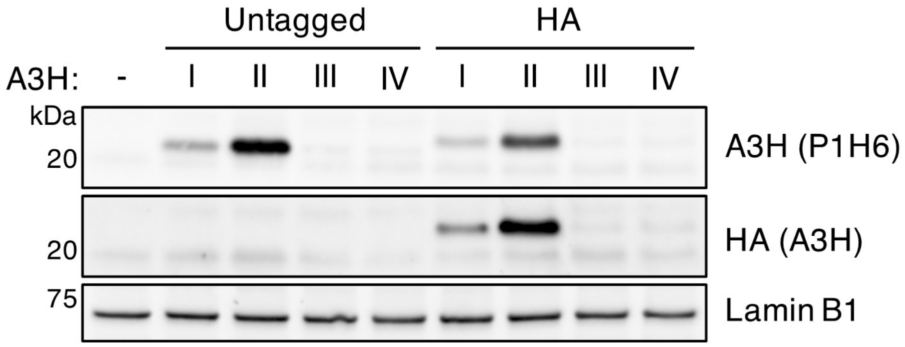

Supplemental Figure S1. The C-terminal HA tag does not alter A3H expression. Immunoblot of the indicated untagged A3H or C-terminally HA-tagged counterparts expressed in 293T cells. Western blotting of whole cell lysates was performed using anti-A3H to detect both untagged and tagged A3H, anti-HA to detect tagged A3H, and anti-Lamin B1 as a loading control.

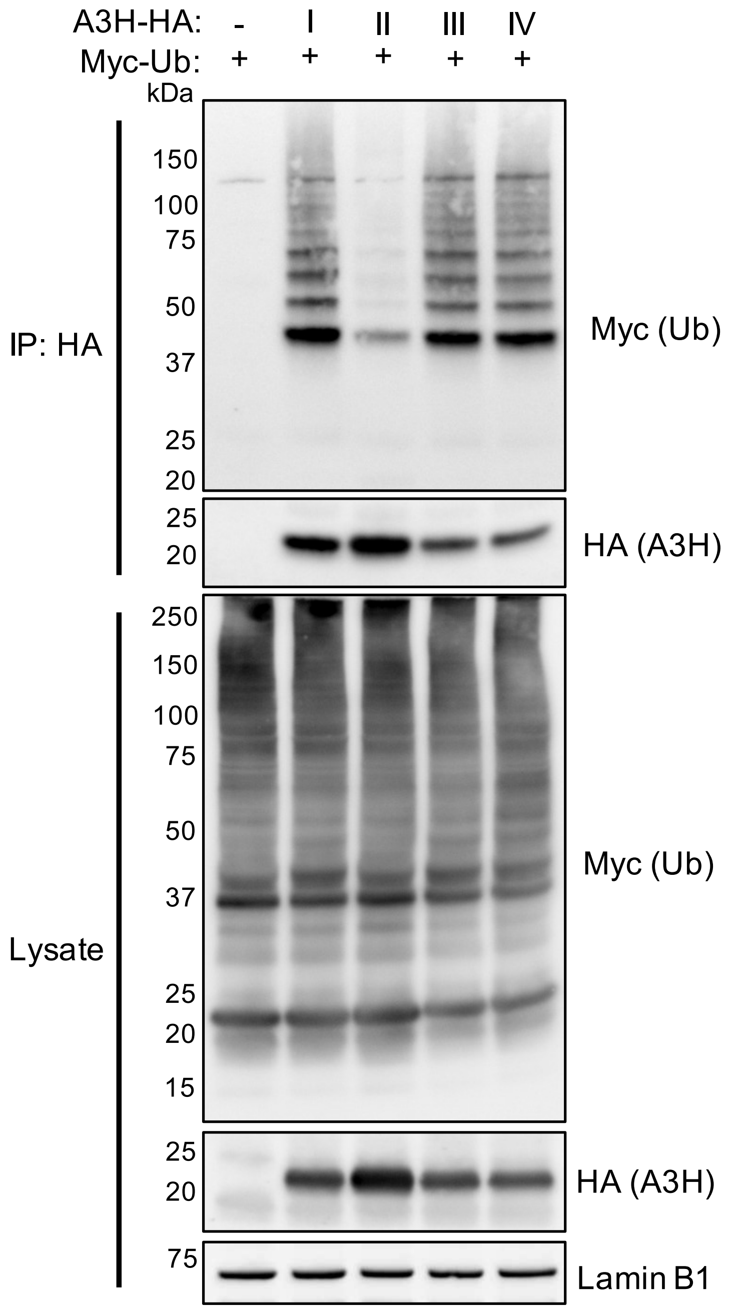

Supplemental Figure S2. Ubiquitination of A3H haplotypes. 293T cells were co-transfected with plasmids expressing A3H-HA haplotypes or vector control, as indicated, alongside myc-tagged ubiquitin (myc-Ub). Whole cell lysates were immunoprecipitated with anti-HA resin, and western blotting was performed with anti-HA and anti-myc antibodies. Western blotting of whole cell lysates was performed using anti-HA to confirm expression of A3H, anti-Myc to detect ubiquitinated proteins, and anti-Lamin B1 as a loading control.
